# Prevalence of SARS-CoV-2 Infection Among COVID-19 Reverse Transcription-Polymerase Chain Reaction (RT-PCR) Laboratory Workers in Bangladesh

**DOI:** 10.7759/cureus.24217

**Published:** 2022-04-17

**Authors:** Mohammad Jahidur Rahman Khan, Samshad Jahan Shumu, Farzana Mim, Ruksana Raihan, Nusrat Mannan, Md. Selim Reza, Nazia Hasan Khan, Arifa Akram, Amirul Huda Bhuiyan, Paroma Deb

**Affiliations:** 1 Department of Microbiology, Shaheed Suhrawardy Medical College, Dhaka, BGD; 2 Department of Biochemistry and Molecular Biology, Jahangirnagar University, Dhaka, BGD; 3 Department of Microbiology, US-Bangla Medical College, Dhaka, BGD; 4 Department of Virology, US-Bangla Medical College, Dhaka, BGD; 5 Department of Infectious Disease, Bangabandhu Sheikh Mujib Medical College, Faridpur, BGD; 6 Department of Infectious Disease, United Hospital Limited, Dhaka, BGD; 7 Department of Virology, National Institute of Laboratory Medicine and Referral Center, Dhaka, BGD; 8 Department of Infectious Disease, Narayanganj 300 Bed Hospital, Narayanganj, BGD; 9 Department of Virology, Dhaka Medical College Hospital, Dhaka, BGD

**Keywords:** cross contamination, laboratory health worker, a risk factor, personal protection equipment, rt-pcr laboratory

## Abstract

Background: Healthcare workers (HCWs) at the frontline are confronting a substantial risk of infection during the COVID-19 pandemic. This emerging virus created specific hazards to researchers and laboratory staff in a clinical setting, underlined by rapid and extensive worldwide transmission.

Objectives: This study aimed to investigate the prevalence of SARS-CoV-2 infection among COVID-19 reverse transcription-polymerase chain reaction (RT-PCR) laboratory health workers in Bangladesh.

Materials and methods: This retrospective study was conducted between October 2 to December 2, 2020. A total of 508 participants, including doctors, scientific officers, medical technologists, and cleaners working in several COVID-19 RT-PCR laboratories, were included in this study. Data were collected from each participant using a semi-structured questionnaire prepared in the format of an anonymous Google form. All statistical analyses were performed using SPSS, version 25.0 (SPSS Inc., Chicago, IL, USA).

Results: Out of the 508 participants, 295 tested positive for SARS-CoV-2 RT-PCR. Among the positive cases, 202 were men, and 93 were women, with a median age of 30 years. The most positive cases were medical technologists (53.22%) followed by doctors (28.8%). Out of the 271 symptomatic positive cases, the most typical symptoms were fever (78.5%), fatigue (70%), loss of smell and taste (65%), and cough (64%). Hypertension, obesity, and diabetes were found in 8.8%, 8.8%, and 7.1% positive cases. A + blood group was present in 37% of the positive cases, followed by the B+ blood group (27%) and O+ blood group (25%). Inadequate supply of personal protective equipment (PPE), absence of negative pressure ventilation, laboratory contamination, and no training on molecular test methods were found in 13.8%, 67.8%, 44.7%, and 40.6% of positive cases, respectively.

Conclusion: Evaluating the infection status of laboratory HCWs is crucial for drawing attention from the public, providing practical suggestions for government agencies, and increasing protective measures for laboratory HCWs.

## Introduction

Since its discovery, SARS-CoV-2 has become a pandemic. As of September 4, 2021, there were 220,362,472 reported cases and 4,562,679 deaths worldwide [1]. In Bangladesh, the first case of SARS-CoV-2 infection was confirmed on March 8, 2020. Subsequently, Bangladesh faced an increasing risk of imports and some local cluster cases of COVID-19. As of September 4, 2021, 1,510,283 confirmed cases and 26,432 deaths in Bangladesh [2].

Health care personnel around the globe have the most significant risk of getting infected and infecting others in their surrounding environment [3]. According to initial estimation, healthcare workers (HCWs) account for 10%-20% of all confirmed cases [4]. During the pandemic, medical services worldwide face an unavoidable burden of public health challenges [5]. In Bangladesh, most molecular laboratories performed RT-PCR to detect SARS-CoV-2 have been established after the pandemic began. These facilities were confronted with an increased amount of real-time reverse transcription-polymerase chain reaction (RT-PCR) testing of SARS-CoV-2 for patients suspected of COVID-19, quarantined HCWs; travellers came back from high-risk countries as well as other required samples. The staff available for the laboratory was swiftly deployed to receive a large number of clinical samples without adequate amounts of training and personal protective equipments (PPEs). To confront this novel coronavirus never experienced before, some public health laboratory workers overlooked concerns about the possible risks of SARS-CoV-2 infection from their occupational exposure. While the protection of laboratory HCWs during the COVID-19 pandemic is one of the primary concerns, data regarding this issue are still inadequate [6].

At present, around 1200 health workers, including doctors, microbiologists, biochemists, molecular biologists, medical technologists, and cleaners, are working in over 100 COVID-19 RT-PCR laboratories across the country [7]. Many of them were infected by SARS-CoV-2 during this ongoing pandemic. As a result, they became a source of SARS-CoV-2 viral spread in a number of laboratories [8]. The testing capacity of a COVID-19 RT-PCR laboratory is reduced when several workers become SARS-CoV-2 infected. The physical environment of the laboratory and workload play an essential role in transmitting SARS-CoV-2 among the laboratory workers [9]. The chance of getting infected by SARS-CoV-2 also depends on a laboratory health worker's age, comorbidity, and functional skill [10].

Thus, we conducted a retrospective study to investigate the prevalence of SARS-CoV-2 infection among COVID-19 RT-PCR laboratory HCWs in Bangladesh and assess the underlying factors related to the high infection rate of SARS-CoV-2.

This article was previously posted to the medRxiv preprint server on December 5, 2021.

## Materials and methods

Study design and data collection

We conducted a retrospective online survey from October 2 to December 2, 2020. A semi-structured questionnaire was prepared using an anonymous Google form. The generated link was shared with the focal persons of each laboratory and several Facebook and WhatsApp groups involving doctors and medical technologists. We decided to collect the data using online approaches and maintain social distance during Bangladesh's pandemic. Additional data were collected from some participants who did not fill out the Google form over the telephone. A hard copy of the questionnaire was also supplied to some participants who were not habituated to online submission by Google form. All participants provided informed consent. Ethical clearance was obtained from the Institutional Ethics Review Committee of Shaheed Suhrawardy Medical College, Dhaka, Bangladesh (protocol: ShSMCH/Ethical/2021/08).

Study sample

A total of 508 laboratory health workers, including doctors, scientific officers (microbiologists, biochemists, and molecular biologists), medical technologists, and cleaners, filled up the Google form. Twenty-six participants were excluded as they had COVID-19-like symptoms, but RT-PCR did not confirm the diagnosis. The remaining 508 laboratory health workers from multiple COVID-19 RT-PCR laboratories were included in this study. Informed consent was obtained from all participants.

Statistical analysis

The confirmed COVID-19 cases among HCWs were categorized according to the following parameters: sex, occupation type, hospital type, infection status, and others. All statistical analyses were performed using SPSS, version 25.0 (SPSS Inc., Chicago, IL, USA).

## Results

Among the 508 participants, 295 (58%) tested positive for SARS-CoV-2 RT-PCR, and 237 (80.3%) were between the 24-44 years age group; male participants were 68.5%, and females were 31.5% (Table 1).

**Table 1:** Demographic data of the study population. PCR: polymerase chain reaction.

Characterstics	Total	SARS-CoV-2 RT-PCR Positive	SARS-CoV-2 RT-PCR Negative
Number (%)	508 (100%)	295 (58%)	213 (42%)
Age (years)			
Median	30	30	30
<24 (%)	50 (9.8%)	27 (9.2%)	23 (10.8%)
24-44 (%)	413 (81.3%)	237 (80.3%)	176 (82.6%)
>44 (%)	45 (8.9%)	31 (10.5%)	14 (6.6%)
Sex			
Male (%)	344 (67.7%)	202 (68.5%)	142 (66.7%)
Female (%)	164 (32.3%)	93 (31.5%)	71 (33.3%)

Most participants were medical technologists (53.7%), followed by doctors (27.2%) (Table 2).

**Table 2:** Infection rate according to the designation of laboratory workers. PCR: polymerase chain reaction.

Designation	Total participants (n= 508)	SARS-CoV-2 RT-PCR	
		Positive (n= 295)	Negative (n= 213)
Doctor	138 (27.2%)	85 (61.6%)	53 (38.4%)
Scientific officer	53 (10.5%)	27 (50.9%)	26 (49.1%)
Medical Technologist	273 (53.7%)	157 (57.5%)	116 (42.5%)
Cleaner	44 (8.6%)	26 (59.0%)	18 (41.0%)

**Figure 1:**
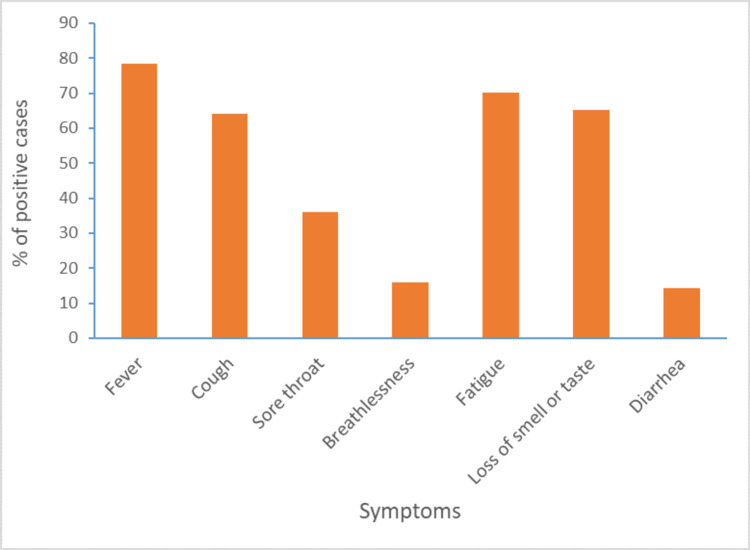
Symptoms of the SARS-CoV-2 reverse transcription-polymerase chain reaction (RT-PCR) positive cases (%).

Among the 295 positive cases, 271 were symptomatic. Analyzing the symptoms, we found 78.5% of them had a fever, fatigue (70%), loss of smell and taste (65%), cough (64%), and others (Figure 1).

**Figure 2:**
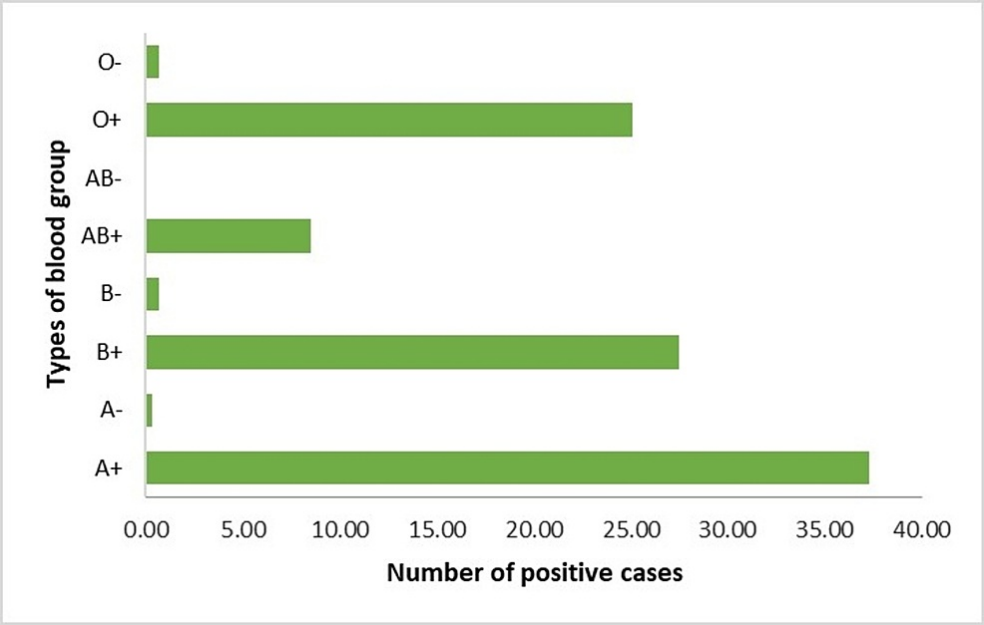
Blood group distribution among the SARS-CoV-2 reverse transcription-polymerase chain reaction (RT-PCR) positive cases.

Among the positive cases, the A+ blood group (37%) was affected more by COVID-19, followed by the B+ blood group (27%) (Figure 2).

We analyzed their comorbidity status and found that hypertension and obesity were most common, 8% in both cases, followed by diabetes (7%) (Table 3).

**Table 3:** Comorbidities found in SARS-CoV-2 RT-PCR-positive and SARS-CoV-2 RT-PCR-negative cases. DM: diabetes mellitus; HTN: hypertension; IHD: ischemic heart disease, RT-PCR: reverse transcription-polymerase chain reaction.

Comorbidity	Total participants (n= 508)	SARS-CoV-2 RT-PCR	
		Positive (n= 295)	Negative (n= 213)
DM	28 (5.5%)	21 (7.1%)	07 (3.3%)
HTN	38 (7.5%)	26 (8.8%)	12 (5.6%)
Asthma	21 (4.1%)	15 (5.0%)	06 (2.8%)
Obesity	44 (8.6%)	26 (8.8%)	18 (8.4%)
IHD	06 (1.1%)	05 (1.7%)	01 (0.5%)

Among the positive cases, 13.8% did not have an adequate supply of PPE, 67.8% did not have the negative pressure ventilation system, 44.7% had an incidence of laboratory contamination, and 40.6% did not receive any training on molecular test methods or quality control (QC) (Table 4).

**Table 4:** Association of risk factors among SARS-CoV-2 RT-PCR-positive cases. PPE, personal protective equipment, RT-PCR: reverse transcription-polymerase chain reaction.

Risk factors	SARS-CoV-2 RT-PCR-positive cases
Inadequate supply of standard PPE	13.8%
Absence of negative pressure ventilation	67.8%
Incidence of laboratory contamination	44.7%
No training on molecular test methods	40.6%

## Discussion

Findings from a previous pandemic of other coronaviruses revealed that frontline HCWs were at the highest risk of infection because of close contact with infected patients, touching the contaminated surfaces, the hiding of epidemiological histories by patients, inadequate training for infection prevention, and control and conducting the high-risk procedures in airway management [11,12]. Additional laboratory professionals, including virologists, microbiologists, medical technologists, and cleaners, are also at high risk through exposure to specimens collected from SARS-CoV-2 infected patients. This study retrospectively collected epidemiological and related data from laboratory personnel working in multiple COVID-19 RT-PCR laboratories. Among the 508 participants of our research, we found that 295 (58%) lab workers became positive during their services, and most of them were male and young (24-44 years age group).

 Among laboratory health workers, medical technologists possess a higher risk of regular handling of both symptomatic and asymptomatic cases [13]. Our study also found that medical technologists affected almost 53% of cases. Analyzing the symptoms of positive cases, we found fever (78%), fatigue (70%), loss of smell and taste (65%), cough (64%), breathlessness (15%), and diarrhoea (14%). Most of the cases were symptomatic (91%). A meta-analysis study on COVID-19 comorbidities shows that the most common comorbidities identified are hypertension (15.8%), which also matched our research; we found it in 8% of cases [14]. Blood group A had a significantly higher risk for acquiring COVID-19 than other blood groups in our study, which is also matched with the study of Barcelona [15].

SARS-CoV-2 can be transmitted during the incubation period when a patient has nonspecific symptoms or no symptoms at all [14]. Therefore, it is necessary to protect them from SARS-CoV-2 infection, and additional transmission-based precautions should be taken [15]. HCWs infected by SARS-CoV-2 can increase the risk of transmission, and their absence from work can decrease health service performance. These may disrupt the chain management of transmission [16]. To minimize the risk of transmission, HCWs should be provided with sufficient PPE supplies, training on infection control, maintenance of personal hygiene, and waste management [17]. Laboratory staff is advised to use PPE like the surgical or N95 mask, gowns, and shield in the correct order, and they must be trained about it. Several studies have suggested that factors such as sufficient supplies of PPE, hands-on training on how to use them, etc., perform a crucial role in controlling such infections, which notably decreases the risk of transmission [18]. During the pandemic, especially at the initial stage, the global scarcity of masks, respirators, face shields, and gowns developed due to the sudden increase in demand and supply chain interference. Therefore, laboratory workers must preserve PPE by increased use or reuse, and infection prevention and control protocols could be maintained for the same reason [19]. Our study revealed that 13.8% of SARS-CoV-2 infected laboratory workers had an inadequate supply of PPE, and 67.8% had no negative pressure ventilation system in their workplace. In addition, 40.6% of SARS-CoV-2 infected laboratory workers did not train on molecular test methods or QC.

HCWs play an essential role in in-hospital transmission. Therefore, they are a potential source of nosocomial infection [20-22]. In SARS-CoV-2 and Middle East respiratory syndrome coronavirus (MERS-CoV) infection, nosocomial outbreaks have played a crucial part in spreading these viruses. The proportions of nosocomial conditions with early outbreaks of COVID-19, SARS, and MERS were 44.0%, 36.0%, and 56.0%, respectively [23]. In our study, 44.7% of SARS-CoV-2 infected laboratory workers gave a history of laboratory contamination within six months.

Our study has some notable limitations. Firstly, health workers may be infected outside the working place. Secondly, we could not collect biochemical data from all HCWs and could not manage the duration of hospital stay. Moreover, we did not obtain whole genome sequencing of the HCWs who tested positive for SARS-CoV-2, and analyses should be interpreted with caution because of the small sample size.

## Conclusions

The safety of laboratory HCWs should be confirmed to end the pandemic, as COVID-19 is ongoing. In this study, we tried to analyze the infection status of laboratory HCWs as it was not done before in Bangladesh; it is also essential to attract enough attention from the government and the public. This study will draw the attention of the government and non-government agencies to maintain the QC of COVID-19 RT-PCR laboratories, improve protective measures like the adequate supply of PPE, and arrange more hands-on training for laboratory health workers.
